# Validation of the EPACODI-1 Scale: University Students’ Perceptions of Inclusive Education

**DOI:** 10.3390/ejihpe13030049

**Published:** 2023-03-20

**Authors:** José-Antonio Morales-Endrino, Jesús Molina-Saorín, José-Antonio Marín-Marín

**Affiliations:** 1 Department of Didactics and School Organisation, University of Murcia, 30100 Murcia, Spain; 2 Department of Didactics and School Organisation, University of Granada, 18071 Granada, Spain

**Keywords:** inclusive education, international convention, perception, training, university students

## Abstract

In the last fifteen years, changes have been taking place in education systems at the international and national levels that aim to achieve, in the near future, the objectives set by the UN International Convention on the Rights of Persons with Disabilities (hereafter CRPD). Spain is no stranger to these objectives, as recognized in the new Organic Law for the Modification of the Law on the Organization of Education. This situation makes it necessary to know the perceptions that professionals in training (about to graduate) from faculties of education have about the legal content established by articles 4.1.i and 24 of the CRPD on inclusive education. In order to ascertain these perceptions, the EPACO-DI-1 instrument was used in a quantitative and confirmatory study by means of a multivariate factor analysis (CFA), applying the parallel estimation method of ordinary least squares (OLS) and principal axes with polychoric correlation and promax oblique rotation. This study involved 552 fourth-year students between the ages of 21 and over 45 from the specializations offered by the Faculty of Education of the University of Murcia. The obtained results are consistent and show the validity of the EPACODI-1 scale for determining the perceptions of professionals in training on inclusive education.

## 1. Introduction

Nearly two decades after the approval of the UN International Convention on the Rights of Persons with Disabilities [1] and almost three lustrums after its ratification by the Spanish state (2008), it would be of significant relevance to try to ascertain the scope that has been achieved in terms of inclusive education in the university education sphere, especially with regard to the training of teaching staff and professionals who work with people who are discriminated against due to low functional performance (art. 4.1.i) and to the degree of compliance with the rights advocated in Article 24 [1].

The transposition of the legal guidelines of the CRPD into the Spanish legislative framework was established (for its development and implementation) with the approval and entry into force of the Royal Legislative Decree [2] for the purpose of responding to and updating the vital and social situations of people that are excluded due to their low functional performance, with the aim of leading them to the full enjoyment—and an equal footing with others—of their human rights as well as universality and non-discrimination in the right to inclusive education.

In the field of education, Article 72.1 of the Organic Law on Education [3] prescribes that the educational administrations shall have teaching staff with the corresponding specialties and qualified professionals as well as the means and materials necessary for the appropriate care of pupils with specific needs. It also provides (in Article 72.4) that they shall promote the training of teachers and other professionals related to the treatment of pupils with special educational needs [3] (p. 17179). Although many legal adaptations have been carried out at all legislative levels, such as the Code of Disability Law [4], the approval and entry into force of the Royal Legislative Decree [3] was significant for the purpose of responding to and updating the vital and social situations of people that are excluded due to their low functional performance, with the aim of leading them to the full enjoyment –on equal terms with others– of their human rights as well as universality and non-discrimination in the right to inclusive education.

For this educational purpose, at the national level, the latest legal amendment referring to education has a precise bearing on achieving inclusive, equitable, and democratic quality education, as this new law is supposed to reaffirm cfr. LOMLOE [5]. A careful reading of this modification of the education law reveals, first of all, that it continues to consider that there may be educational needs that acquire the status of special needs; as if this delirium were not enough, it also indicates that, in addition, pupils with special educational needs (SIC) are but a subcategory within another (broader) category called pupils with specific educational support needs (art. 71). This new aesthetic change to the law (already dreary as far as inclusion is concerned, with hardly any progress in this area since 1990) does not amend the brake that the previous regulatory text [5] had already put on the implementation of the right to quality education, which (until it is otherwise stated) also applies to these students with special educational needs, in particular (as well as the rest, in general), as prescribed by [6] and indicated by [7]. However, an analysis of the current Article 74.1 of the LOMLOE [5] shows that it continues to maintain the possibility that some pupils may continue to be educated in so-called special education centers, which would contravene compliance with the aforementioned article 24 of the CRPD [1] insofar as it is a reason for discrimination against these pupils, making this section a discriminatory provision that is completely contrary to the right to inclusive, equitable, and quality education established by the CRPD, protected by our Constitution, and paradoxically contained in the preamble of this new law [5].

In this sense, [7] reminds us that our ethics and professionalism should make us feel —without further delay— obliged to comply with these regulations. This new law [5], in its fourth additional provision (on the evolution of the schooling of pupils with special educational needs) makes its defense of special education centers crystal-clear (ignoring the CRPD):

…the education authorities shall continue to provide the necessary support to special education centers so that, in addition to providing schooling for pupils requiring highly specialized attention, they can act as reference and support centers for mainstream schools.

[5] (p. 122942)

In this way, as impertinent as it is contrary to the guidelines dictated by the CRPD, the possibility of segregating part of the student body continues to be maintained, especially those with lower functional performance, especially at an intellectual level, as stated by numerous specialists [7,8,9,10,11].

Therefore, if we take into account that training is a fundamental pillar for creating inclusive, fair, equitable, and democratic societies, as stated by [12,13], among others, it is reasonable to think that all the training knowledge acquired by future teachers in this area (as education professionals) will have a fundamental influence on the development and subsequent practice of their profession, as stated by, among others, [9,10,11,14,15,16], which is why this research has been carried out.

## 2. Materials and Methods

This research is part of a quantitative and confirmatory study [17,18] using multivariate methods, specifically a confirmatory factor analysis (CFA) applying the parallel method of ordinary least squares (OLS) and unweighted least squares (ULS) with polychoric correlation and promax oblique rotation, with the aim of determining and analyzing the perceptions, beliefs, and attitudes shown by final-year university students (about to graduate) of the Faculty of Education of the University of Murcia with respect to the inclusive education prescribed by art. No. 24 of the CRPD and the rights of people who are discriminated against due to low functional performance. The sample was made up of fourth-year students enrolled in the training specialties taught by the aforementioned faculty, as can be seen in the descriptive tables (1 and 2). The information was collected using a quantitative instrument (in digital and printed versions) called EPACODI-1, a scale of university students’ perceptions of the training received on the UN International Convention on the Rights of Persons with Disabilities, which was created ad hoc following the recommendations of, among others, [19,20,21]. The creation and design of the research instrument were based on the EPSD-1 scale [16].

For the creation of the scale, the prospective Delphi method was followed (cf. [22,23,24,25]), where the scale was submitted to the judgement of expert judges in various disciplines through a group communication process that is effective in allowing the assessment of a group of individuals considered as a whole (maintaining anonymity among them). Through several rounds, they can deal with a complex problem. By email, they were sent the form with the criteria on the basis of which their expert judgement should be carried out, with a detailed description of each criterion (clarity, relevance, coherence, and pertinence (CRCP)). Based on these criteria, the judges had to express their assessment after several prior internalization readings.

The EPACODI-1 scale is a Likert-type scale with five response levels (coded with the following values: 5—strongly agree; 4—agree; 3—don’t know/not sure; 2—disagree; and 1—strongly disagree). All items are developed in five blocks, the first block (block 1) is composed of 9 items referring to socio-demographic data. The second block is dedicated to the concept of a person with a disability, using qualitative and quantitative indicators. The third block is made up of 14 items that inquire into the content of the CRPD. The fourth block contains 20 items focusing on the training received during the degree in reference to the rights recognized in the convention. Finally, block 5 is made up of 25 items focusing on the assessment of the rights of people who are discriminated against on the grounds of low functional performance.

In order to obtain the data, the guidelines established by the Research Ethics Committee of the University of Murcia and the laws on personal data protection of the participants were followed.

Once the scale had been applied, the obtained data were processed using [26], and the information was subsequently exported to the statistical programs [27,28,29,30], with which the research instrument was tested. The results reported by each statistical program were compared with each other to verify that the results obtained by each of these statistical programs were in agreement.

In this article, the results of the validation of four factors of the EPACODI-1 scale are presented with the intention of investigating the following specific research objectives:

OB1: analyze the personal perceptions of university students regarding the rights of people with low functional performance.
OB2: assess the training received on the right to educational inclusion of students with a higher degree of disability, according to the CRPD.
OB3: discover the perceptions of university students on situations of discrimination due to low functional performance.
OB4: determine the perceptions of university students on the possibility of raising awareness and training (at institutional level), for both the teaching staff and the faculty’s governing team, with regard to the rights recognized in the CRPD.

As mentioned above, the following tables (Table 1 and Table 2) present the descriptive statistics of the sample.

As can be seen (Table 1), the obtained sample (552 participants) represented 56.33% of the total population (N = 980), with a confidence level of 95% and an estimation error of 3% (19.5% male and 80.5% female). The ages of the participants ranged from 21 years to over 45 years. 

The following table (Table 2) presents the different specialties of the participants and the percentages of participation for each specialty.

As can be seen in Table 2, the highest percentage of participants belonged to the specialty of hearing and language (13.8%), with the lowest percentage coming from the specialty of educational resources for school and leisure time (4.0%).

## 3. Results 

The validation of the EPACODI-1 scale was based on the application of the multivariate factorial method (as recommended by [19,20,21]) as well as the rational equivalence method (through the calculation of Cronbach’s α [31]). Table 3 presents the validity and reliability data of the EPACODI-1 scale, which were obtained by applying various methods.

As can be seen in Table 3, an evaluation of the validity of the EPACODI-1 scale was carried out by the expert judges using Kendall’s Ŵ coefficient, reaching a value of 0.962, showing satisfactory validity. The reliability of the instrument was tested using the split-half method and the Kuder–Richardson method (inter-form correlation of 0.988), obtaining values of 0.994 for the Spearman–Brown coefficient and 0.987 for the Guttman coefficient, which shows the high internal consistency of the instrument. A reliability analysis was also carried out using the rational equivalence method, obtaining a Cronbach’s α of 0.931, which implies that the scale has a high level of reliability.

**Figure 1:**
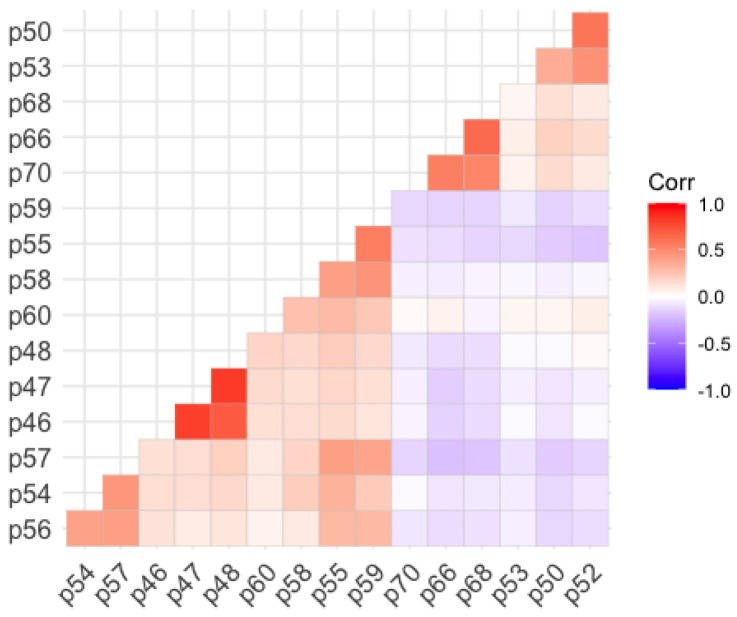
Polychoric correlation matrix. Note: the data were obtained using R (version 1.3.1056).

Subsequently, a confirmatory factor analysis was applied, using the parallel ordinary least squares (OLS) and unweighted least squares (ULS) method as the estimation method, following the recommendations of [32,33], based on the polychoric correlation matrix, which is more appropriate for ordinal variables. The following graph (Figure 1) shows the polychoric correlation matrix of the studied variables with their significance levels. A Kaiser–Meyer–Olkin (KMO) test confirmed the relevance of the factor analysis, obtaining an MSA value of 0.75 (a percentage considered suitable for interpretation). As for Bartlett’s test of sphericity (as can be seen in Figure 1), it showed that the matrix of polychoric correlations complied with the condition of the intercorrelation of the items, with *X^2^* = 635.28 (gl = 62 and *p* < 0.001). Together, the retained factors explained 65% of the total variance.

The MSA values for each variable are presented in Table 4.

**Figure 2:**
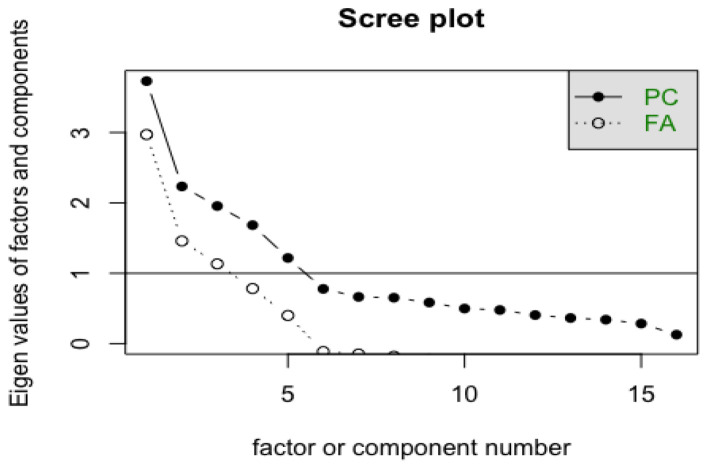
Sedimentation graph. Note: This figure shows a sedimentation graph with the eigenvalue levels of the factors and their components. The data were obtained using R (version 1.3.1056).

**Figure 3:**
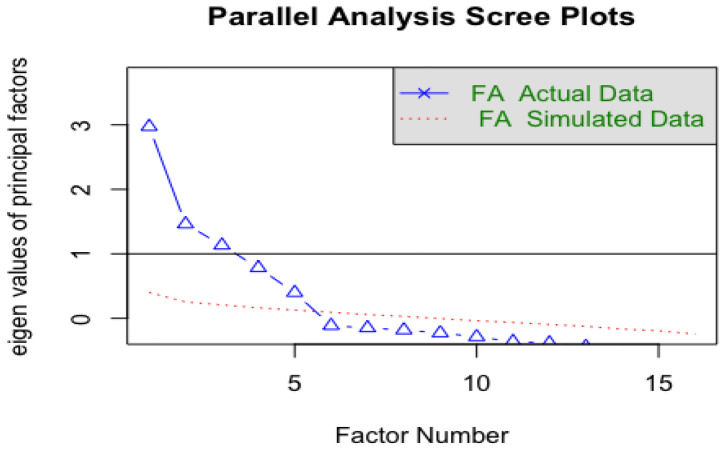
Sedimentation graph obtained by parallel analysis. Note: Figure 3 shows a sedimentation plot of the factor components after a parallel analysis for the retention of the four delimited factors (the fourth factor is at an eigenvalue somewhat below 1 (approx. 0.80). The data were obtained using R (version 1.3.1056).

The following graphs (Figure 2 and Figure 3) show the information that was reported to obtain the four factors delimited by the scree plot and the parallel analysis scree plots that recommended the delimitation of the four factors that were finally retained.

**Figure 4:**
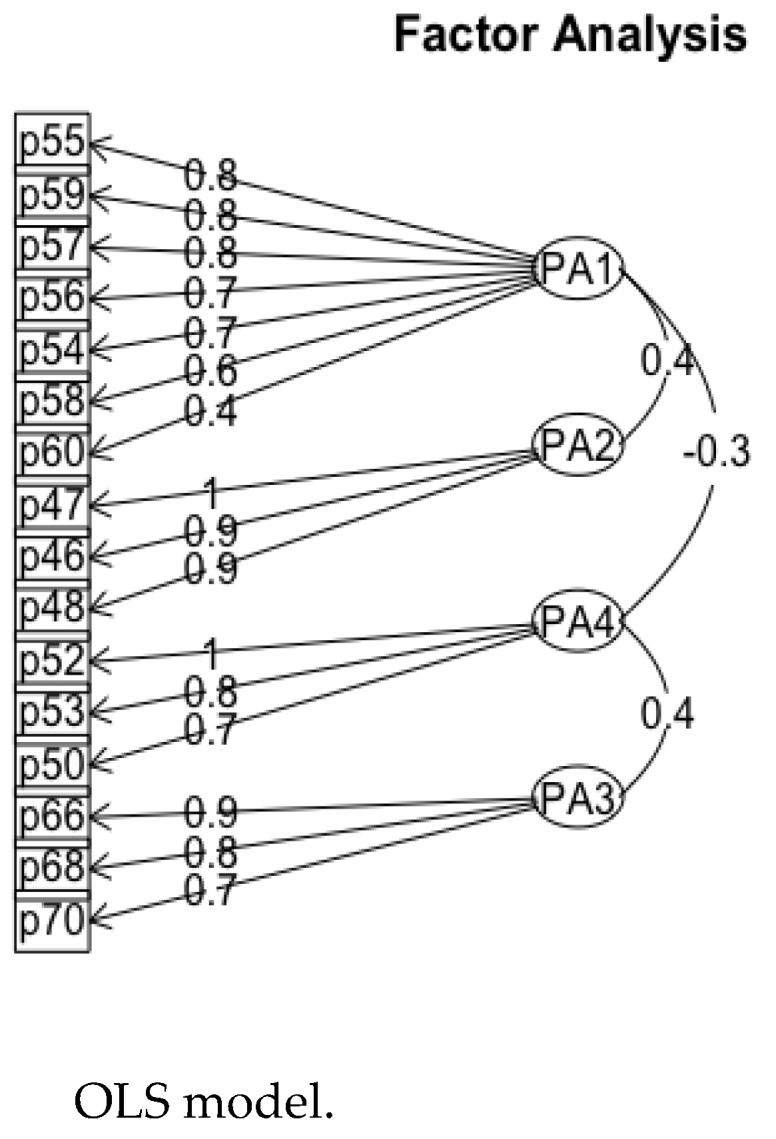
Plot of the factorial model of the four retained factors. Note: This is a diagram of the factorial model with the four factors delimited, showing the communal value of each variable to its factor. The data were obtained using R (version 1.3.1056).

By applying promax oblique rotation, the obtained OLS (ordinary least squares) model suggested the retention of these four factors. A diagram of the resulting model is shown in the figure below (Figure 4):

The following table (Table 4) shows the standardized loadings (standardized matrix) based on the polychoric correlation matrix after the promax rotation.

Table 5 and Table 6 present the grouping of the four factors with their variance loadings and a correlation matrix with their correlational significance levels.

As the matrix of the factorial model revealed, the variables were grouped into the four retained factors, which were well defined, obtaining an RMSR (standardized root-mean-square residual) of 0.04, which reported a good goodness of fit of the scale, according to the parameters established by [34,35]. Table 7 presents the fit indices of the scale that were retained by the polychoric correlation.

As can be seen in Table 7, the values reported for these four factors indicate that the goodness of fit of the analyzed scale is adequate. If Pearson’s correlation is applied, considering the data to be normalized by the large number of participants (*n* = 552), the goodness-of-fit indices obtained for these factors can be considered to be quite satisfactory, as shown in Table 8.

With respect to the four retained factors, they are structured as follows: The factor FPA1/PA2 groups the variables p54 to p60 and was named the personal perception of the rights of people with low functional performance. This factor explained 22% of the total variance. 

It is worth noting that as far as this factor (FPA1/PA2) is concerned, the values reveal that it obtained a good reliability statistic (α = 0.693), and its descriptive statistics (M = 2.78; SD = 0.965, *p* < 0.001) reveal that the respondents gave it a value somewhat below the mean in such a way that it allows us to verify the existence of disagreement in terms of personally perceiving symptoms of discrimination in some aspects of the rights of people who present low functional performance, both in the university environment and in their social environment.

As for the factor FPA2/PA5, this factor groups variables p46, p47, and p48 and was labeled the perception of the most appropriate schooling for students with low functional performance. This variable explained 16% of the total variance. This factor revealed a high alpha value (α = 0.917), but in terms of the mean, this factor yielded the same value as the previous one (M = 2.78) but with a higher standard deviation (SD = 1.028, *p* < 0.001), showing that the respondents gave lower scores with respect to the most appropriate type of schooling for students with low functional performance.

As for the factor FPA4/PA3, this factor groups the variables p50, p52, and p53 and was named the perception of discrimination situations due to low functional performance. This factor explained 14% of the total variance. The analysis revealed that the respondents gave the highest score to this factor (α = 0.679, M = 4.72; SD = 0.583, *p* < 0.001). This fact demonstrates that the respondents did not perceive prejudice among their contacts and friends related to situations of discrimination on the grounds of low functional performance.

The last factor that was analyzed, FPA3/PA7, groups the variables p66, p68, and p70 and was called the awareness of training (at the institutional level and among teachers and the government team) with respect to the rights recognized in the CRPD. This variable explained 13% of the total variance. The analysis revealed that the respondents also placed a high value on this factor (α = 0.699, M = 4.10; SD = 0.892, *p* < 0.001) in relation to the fact that faculty and staff should be trained and aware of the rights recognized in the CRPD [1].

With regard to the results of the types of correlations between the factors, the factor FPA1/PA2, the personal perception of the rights of people with low functional performance, presented a statistically significant direct correlation (*p* = 0.36) with the factor FPA2/PA5, which allows us to interpret that when there is an increase in the perception of the most appropriate schooling for students with low functional performance, there is an increase in the personal perception of the rights of people with low functional performance.

On the other hand, the factors FPA4/PA3 and FPA3/PA7 were also directly correlated (*p* = 0.44), so it can be interpreted that an increase or decrease in the perception of situations of discrimination due to low functional performance may imply an increase or decrease in the awareness of training (at the institutional level and among teachers and the government team) regarding the rights recognized in the CRPD. In addition, the factors FPA1/PA2 and FPA4/PA3 showed a statistically significant inverse correlation (*p* = −0.32), so it can be interpreted that a decrease in the personal perception of the rights of people with low functional performance may imply a decrease in the perception of situations of discrimination due to low functional performance.

FPA1/PA2 and FPA3/PA7 also showed a statistically significant inverse correlation (*p* = −0.26), so it can be concluded that a decrease in the personal perception of the rights of people with low functional performance may imply a decrease in the awareness of training (at the institutional level and among teachers and the government team) regarding the rights recognized in the CRPD.

## 4. Discussion

By analyzing the specialized literature [34,36,37,38,39], the extraction of factors was carried out following the ordinary least squares (OLS) methods and the method of principal axes, as recommended in [20] for studies in which there is an absence of normality. The matrix of polychoric correlations was precisely analyzed. To determine the number of factors, several criteria present in the specialized literature were followed [40,41,42,43]. One of the criteria followed was the Gutman–Kaiser rule, where factors with eigenvalues greater than unity (obtained from a parallel analysis) were retained, revealing the existence of four well-defined factors. Although there was critical evidence in the specialized literature regarding this criterion for providing imprecise results, which can compromise the explanatory capacity of a factorial solution (especially when the number of variables is very large or very small), as indicated in [42,43], in the case of this scale, if one looks at the sedimentation plot reported by the analyses that were carried out, the Gutman–Kaiser rule was applied with an eigenvalue somewhat lower than unity (approx. 80), retaining a factor set before the inflection point of the plot, since this is a recommendable option to obtain results reported by the scale with more coherent precision. The variance explained by the model was also taken into account, considering whether the percentage of explained variance was close to or within the ranges recommended in the specialized literature, in which a minimum total explained variance of 65% was presented as a threshold for the extraction of factors [43,44]. Another criterion applied for the retention of factors was to eliminate variables with intercorrelations lower than >0.30.

The relevance of retaining these four factors was also verified since the measurement systems analysis (MSA) reported a value of 0.75, indicating good linearity of the measurement system, and Bartlett’s test of sphericity showed that the matrix of polychoric correlations (for studies lacking normality) complied with the condition of the intercorrelation of the items, with X^2^ = 635.28 (gl = 62 and *p* < 0.001).

Regarding the type of rotation that was applied (promax oblique rotation), the criteria suggested by [41,42,45,46] were followed since these rotations report accurate and reproducible solutions. In [42], the authors indicated that oblique rotation provides estimators close to zero (similar to those of orthogonal rotation), allowing a researcher to delve deeper into the origins of the concepts involved in the factors [11].

According to the evaluations obtained by the factors retained after the analyses applied the goodness of fit to the EPACODI-1 scale in relation to the goodness-of-fit index by means of polychoric correlation (goodness-of-fit index (G.F.I.) = 0.99; adjusted goodness-of-fit index (A.G.F.I.) = 0.81, and root-mean-square residual (R.M.S.R.) = 0.04) and by means of a Pearson correlation (G.F.I. = 0.99; adjusted goodness-of-fit index (A.G.F.I.) = 0.97, and root-mean-square residual (R.M.S.R.) < 0.05), it was revealed that it has good psychometric attributes to provide a clear and accurate picture of the perception of the surveyed trainees with regard to the objectives set for this research, specifically with regard to inclusive education (art. 24 of the CRPD). The EPACODI-1 scale matrix with the name of each factor retained and the percentage of the value contributed by each variable to its factor is presented in Appendix A.

## 5. Conclusions

The study highlights that despite the enormous amount of time that has passed since the ratification of the CRPD by Spain in 2008, the advances in terms of inclusive education advocated by the CRPD [1] have been few and lax (in some aspects) in terms of its implementation in the practical university training environment, and these results are in line with other studies related to the educational inclusion of people that are discriminated against due to low functional performance at the university level, as shown by [7,8,9,10,11,12,13,14,15,16,17,18,19,20,21,22,23,24,25,26,27,28,29,30,31,32,33,34,35,36,37,38,39,40,41,42,43,44,45,46,47,48], among others [14,16,49,50,51,52]. This information contributes greatly to the development of the proposed research objective (OB1) to analyze the personal perception of university students regarding the rights of people with low functional performance.

With regard to the factor FPA2/PA5, the perception of more appropriate schooling for low functioning students, it is noteworthy to say that the respondents had a positive perception towards inclusive education for these students (low functioning) in ordinary public education centers, as their responses to variables V46, V47, and V48 were at levels one and two (totally disagree and disagree), with values against the inclusion of pupils with low functional performance in special education centers (62. 5% for the infant stage, 58.9% for the primary stage, and 55.8% for the secondary stage).

What is noteworthy as well as worrying with respect to factor F2/PA5 is the existence of 17.7% of respondents who responded with levels four and five, stating that they agreed or totally agreed with schooling these students in special education centers, with some of these results being in line with those obtained by other researchers [11,16,53]. This fact reinforces the perception of the lack of training received on the rights recognized in the CRPD since it is quite contradictory that a majority of respondents (59.1%) are in favor of the educational inclusion of students with low functional performance in general education centers yet a high percentage also state that students with low functional performance should be schooled in special education centers (17.7%), surely a direct consequence of a lack of training on the convention. Through these statements, we responded to the proposed research objective (OB2) to assess the perception of the most appropriate schooling for students with low functional performance.

With regard to factor FPA4/PA3, the perception of situations of discrimination due to low functional performance, it should be noted that 96. 2% of respondents positioned themselves in response levels four and five, in agreement or total agreement in not perceiving prejudice among their contacts and friends related to the knowledge that a friend or relative is in a situation of disability, in line with other studies [14,16,47,49,54,55]. These results obtained by the study shed light on the proposed research objective (OB3) to discover university students’ perceptions of situations of discrimination due to low functional performance.

With regard to the last factor, FPA3/PA7, the awareness of training (at the institutional level and among teaching staff and the government team) with respect to the rights recognized in the CRPD, it is considered that 92. 8% of the respondents positioned their answers at levels four and five, stating that they agreed or totally agreed that the faculty and the faculty’s governing body should be trained and aware of the rights recognized in the CRPD [1], which are relevant and applicable to the university environment [11,14,50,50,51]. In this sense, the data shed light on the proposed research objective (OB4) to determine the perceptions of university students regarding the possibility of raising awareness and training (at the institutional level) both the teaching staff and the faculty’s governing team with respect to the rights recognized in the CRPD.

As it has been verified that these factors of the EPACODI-1 scale interrelate to configure the perception of this sample of professionals in training (from the Faculty of Education of the University of Murcia) on their scarce training or total lack of training with respect to the recognized rights of people discriminated against at the educational level on the grounds of low functional performance, all of this is influenced by the lack of training and awareness of the teaching staff and the faculty’s governing body, and this lack of training contributes to increasing the possible prejudices of those surveyed towards people with low functional performance [11,14,47,50,54,56]). Therefore, this clearly shows that the respondents were not aware of the legal contents present in articles 4. 1.i and 24 of [1]; the articles of [3]; the contents of the new education law [5]; the contents of [57], which –on the right to inclusive education– were raised by the UN Committee on the Rights of Persons with Disabilities; or the much-vaunted [58], especially article 4.

It is important to highlight that the main limitation of this study lies in its limitation to the spatial and geographical scope of the Faculty of Education of the University of Murcia. For this reason, in the future, it is planned to give continuity to the use of the EPACODI-1 scale, [11], extending its application and data collection to other faculties of education in Spain and even enabling its application and use in the Latin American geographic area, as it is able to effectively observe the regulations with the highest rank of law at the international level with respect to people with low functional performance, such as the CRPD [1].

## Figures and Tables

**Table 1:** Descriptive statistics of the sample.

Sample	P.M.T.%	Population	N.C.-E.E.	Men	Females	Age: Between 21 and 44 Years	Age: 45 Years and Older
*n =* 552	56.33%	N = 980	95%−3%	19.5%	80.5%	98.4%	1.6%

Note: this is an elaboration of data we obtained using SPSS v.22 and STATS v.2.0.

**Table 2:** Qualifications of participants and contribution % of each specialty.

Degree Being Studied	Percentage of Participants
Early Childhood Education	13.0
Social Education	11.2
Pedagogy	13.2
Primary Education, including:	
Hearing and Language	13.8
Music	6.5
Specific Educational Support Needs	8.5
Physical Education	5.4
Intercultural Education and Learning Difficulties	9.8
Educational Resources for School and Leisure Time	4.0
English	8.3
French	6.3
Total: *n* = 552	100.0

Note: this is an elaboration of data we obtained using SPSS (version 22).

**Table 3:** Validity and reliability analysis of the EPACODI-1 scale.

Kendall’s Ŵ (Judges)	Kuder–Richardson	Spearman–Brown	Guttman	Cronbach’s α	MSA and Bartlett’s Sphericity
0.962	0.988	0.994	0.987	0.931	MSA = 0.92; *X*^2^ = 24,277.78; gl = 903; *p* < 0.001

Note: this is an elaboration of data we obtained using SPSS (v.22) and R (v.4.2.0).

**Table 4:** Matrix of the obtained factorial model and the value of each variable.

Fa/Var	FPA1/PA2	FPA2/PA5	FPA4/PA3	FPA3/PA7	h2	u2	Com.
p46	−0.03	0.86	−0.05	−0.02	0.74	0.256	1.0
p47	0.09	1.01	−0.07	−0.01	0.98	0.021	1.0
p48	0.09	0.86	0.05	−0.02	0.79	0.206	1.0
p50	−0.03	−0.06	0.73	0.08	0.61	0.385	1.0
p52	0.00	0.02	0.98	−0.07	0.89	0.109	1.0
p53	−0.01	−0.03	0.79	−0.09	0.58	0.417	1.0
p54	0.74	0.05	−0.11	0.01	0.64	0.358	1.1
p55	0.82	−0.04	−0.10	0.02	0.71	0.293	1.0
p56	0.74	−0.03	−0.06	−0.13	0.63	0.365	1.1
p57	0.77	−0.01	−0.11	−0.16	0.76	0.244	1.1
p58	0.55	0.00	0.05	0.08	0.28	0.720	1.1
p59	0.80	−0.12	−0.01	−0.05	0.61	0.392	1.1
p60	0.41	0.09	0.13	0.08	0.18	0.823	1.4
p66	0.04	−0.05	−0.01	0.92	0.83	0.174	1.0
p68	−0.02	−0.03	−0.11	0.83	0.64	0.357	1.0
p70	−0.01	0.03	0.03	0.67	0.47	0.531	1.0

h2: communalities, u2: specificity. Note: this is an elaboration of data we obtained using R (v.4.2.0).

**Table 5:** Loadings for each of the four retained factors and their explained variances.

FACTORS	FPA1/PA2	FPA2/PA5	FPA4/PA3	FPA3/PA7
SS loadings	3.57	2.51	2.21	2.06
Variance ratio	0.22	0.16	0.14	0.13
Cumulative variance	0.22	0.38	0.52	0.65
Explained variance	0.34	0.24	0.21	0.20
Cumulative variance	0.34	0.59	0.80	1.00

Note: this is an elaboration of data we obtained using R (v.4.2.0).

**Table 6:** Matrix of correlations of the four retained factors.

FACTORS	FPA1/PA2	FPA2/PA5	FPA4/PA3	FPA3/PA7
FPA1 (PA2)	1			
FPA2 (PA5)	0.36	1		
FPA4 (PA3)	−0.32	−0.10	1	
FPA3 (PA7)	−0.26	−0.17	0.44	1

Note: this is an elaboration of data we obtained using R (v.4.2.0).

**Table 7:** Fit indices of the four factors retained by the polychoric correlation.

Goodness-of-fit index (G.F.I.)	=0.99
Adjusted goodness-of-fit index (A.G.F.I.)	=0.81
Root-mean-square residual (R.M.S.R.)	=0.04

Note: this is an elaboration of data we obtained using R (v.4.2.0).

**Table 8:** Fit indices of the factors analyzed by the means of the Pearson correlations.

Goodness-of-fit index (G.F.I.)	=0.99
Adjusted goodness-of-fit index (A.G.F.I.)	=0.97
Root-mean-square residual (R.M.S.R.)	<0.05

Note: this is an elaboration of data we obtained using R (v.4.2.0) and Factor (v.10.3.01 XP).

## Data Availability

All data from this research are protected and held by the principal investigators. The data will be made available to those interested in this study.

## Appendix A

**Table A1:** Matrix of the EPACODI-1 scale, with the name of each retained factor and the percentage of the value contributed by each variable to its factor.

Factor	Personal Perception of the Rights of People with Low Functional Performance	FPA1/PA2
Var.	Item	%
p33	54. In my view, people with disabilities should not have the same rights as other citizens.	0.79
p34	55. To be honest, I would not want my child’s teacher to have a disability.	0.86
p35	56. From my point of view, people with disabilities should have fewer rights than other citizens.	0.81
p36	57. To be honest, I would not want my child to share a classroom with other students who have a disability.	0.84
p37	58. I think that parents should have the right to know if their child’s teacher has a disability.	0.76
p38	59. If I had a choice, I would never take my child to a pediatrician with a disability.	0.78
p39	60. I must admit that, on occasion, when I have seen a child with cerebral palsy and his parents pushing the pram, I have come to feel a little sorry for them.	0.71
p40	61. I must admit that, on occasion, when I have seen a blind person in the street, I have felt a little sorry for them.	0.68
**Factor**	**Perception of the most appropriate schooling for students with low functional performance**	**FPA2/PA5**
**Var.**	**Item**	**%**
p27	46. In my opinion, for the early childhood education stage, it seems to me that it is appropriate for pupils with a greater degree of disability to be placed in special education centers.	0.89
p28	47. In my opinion, for the primary education stage, it seems to me that it is appropriate for pupils with a greater degree of disability to be placed in special education centers.	0.71
p29	48. In my opinion, for the secondary education stage, it seems to me that it is appropriate for pupils with a higher degree of disability to be placed in special schools.	0.79
**Factor**	**Perception of discrimination situations due to low functional performance**	**FPA4/PA3**
**Var.**	**Item**	**%**
p30	50. In general, I have no problem (or would have no problem) in interacting with people with disabilities on a regular basis.	0.75
p31	52. I would not mind if any of my friends had a disability.	0.66
p32	53. I would not mind if my contacts knew that I have a close relative with a disability.	0.77
**Factor**	**Awareness of training (at institutional level and among teachers and the government team) with respect to the rights recognized in the CRPD**	**FPA3/PA7**
**Var.**	**Item**	**%**
p41	66. I think that, in all the degrees offered by the Faculty of Education, there should be a subject in which students learn everything related to disability and the content of the CRPD.	0.72
p42	68. I consider it relevant, necessary, and non-negotiable for all university students to know, in depth, the rights of persons with disabilities, as developed in the CRPD.	0.72
p43	70. I consider that the university should have a strategic plan on disability (just as it has a strategic plan on gender equality, for example).	0.72

Note: The matrix was obtained from Morales and Molina (in press).
